# Manipulation of light spectrum is an effective tool to regulate biochemical traits and gene expression in lettuce under different replacement methods of nutrient solution

**DOI:** 10.1038/s41598-023-35326-x

**Published:** 2023-05-26

**Authors:** Hamid Reza Soufi, Hamid Reza Roosta, Piotr Stępień, Khalil Malekzadeh, Mohsen Hamidpour

**Affiliations:** 1 Department of Horticultural Sciences, Faculty of Agriculture, Vali-e-Asr University of Rafsanjan, Rafsanjan, Iran; 2 Department of Horticultural Sciences, Faculty of Agriculture and Natural Resources, Arak University, Arāk, Iran; 3 Department of Plant Nutrition, Institute of Soil Science, Plant Nutrition and Environmental Protection, Wroclaw University of Environmental and Life Sciences, ul. Grunwaldzka 53, 50-357 Wrocław, Poland; 4 Department of Genetics and Plant Production, Faculty of Agriculture, Vali-e-Asr University of Rafsanjan, Rafsanjan, Iran; 5 Department of Soil Science and Engineering, Faculty of Agriculture, Vali-e-Asr University of Rafsanjan, Rafsanjan, Iran

**Keywords:** Biochemistry, Physiology, Plant sciences

## Abstract

The use of light-emitting diode (LED) technology represents a promising approach to improve plant growth and metabolic activities. The aim of this study was to investigate the effect of different light spectra: red (656 nm), blue (450 nm), red/blue (3:1), and white (peak at 449 nm) on biochemical properties, photosynthesis and gene expression in two lettuce cultivars (Lollo Rossa and Lollo Bionda) grown under different methods of nutrient solution replacement in hydroponics. Complete replacement and EC-based replacement of nutrient solution increased content of proline and soluble sugars and activity of antioxidant enzymes (CAT, GPX and SOD) under the red/blue LED and red LED light treatments in both cultivars. In addition, the red/blue and the monochromatic red light increased the soluble protein content and the antioxidant activity in the Lollo Rosa cultivar under the replacement method according to the needs of the plant. An increase in flavonoid content in the EC-based method in the Lollo Rosa variety treated with a combination of red and blue light was also observed. The red/blue light had the greatest induction effect on anthocyanin content, expression of the UFGT, CHS, and Rubisco small subunit genes, and the net photosynthetic rate. Data presented here will directly contribute to the development of nutrient solution and LED spectrum management strategies to significantly improve plant growth and metabolism, while avoiding water and nutrient waste, and environmental pollution.

## Introduction

Today, water is a basic human need, and a valuable natural resource^1^. Agriculture, as the largest consumer of water, is the most affected during drought disasters^2^. In this situation, scientists are looking for solutions to reduce water consumption in agriculture. One of them is hydroponic cultivation of crops, and the floating raft system is one of the hydroponic systems for the production of vegetable crops, especially lettuce. In addition to better water use efficiency, hydroponic systems, when operated in a controlled environment, can provide high quality and quantity vegetable production^3^.

However, limited hydroponic cultivation in some countries is due to several reasons, including the high costs of initial tests to determine the amount of elements, EC, pH, the need for specialised labour, and new products and appropriate techniques to control pests and diseases^4^. Maintaining the correct nutrient ratio and electrical conductivity of the nutrient solution at different growth stages is essential for achieving high quality production. The rate of absorption of different ions by the root system is not equal, and elements such as ammonium, nitrate and potassium are absorbed rapidly, while calcium, magnesium and sulphate ions are absorbed slowly and therefore accumulate in solution^5,6^. Some researchers recommend determining the concentration of each element during the period of nutrient solution use, and, depending on the amounts absorbed, adding a replenishment solution containing all or some of the elements depleted from the nutrient solution^5^. Ding et al.^7^ reported that too high and too low EC decreased the soluble sugar content of pakchoi plant (*Brassica campestris* L. ssp. Chinensis), while showing a significant increase in antioxidant enzyme activities. In another study, higher antioxidant content and antioxidant capacity were observed in lettuce even when a concentration of 1/4 of the nutrient solution was applied^8^.

Numerous horticultural crops have been grown in hydroponics in closed growth chambers equipped with novel light source such as light-emitting diodes (LED), referred to as Plant Factory with Artificial Light (PFAL)^9,10^. Manipulations in the LED light environment, such as establishing specific light modes by integrating spectral quality and intensity, can increase crop productivity. It has been reported that different red/blue LED light ratios result in a species-dependent response in growth parameters such as fresh and dry matter and leaf optical indices such as chlorophyll, flavonoid, anthocyanin and carotenoid content^11^. As pointed out by Rehman et al.^12^, irradiation with red light decreased the proline content and the activities of superoxide dismutase and peroxidase in ramie plant (*Boehmeria nivea* L), whereas blue light increased the activities of these enzymes. Recently, red LED light has been reported to increase total phenolic content and radical scavenging activity in *Rubus hongnoensis*, while plants treated with white LED light were characterized by higher superoxide dismutase (SOD), catalase (CAT), and ascorbate peroxidase (APX) activities^13^.

Anthocyanins belong to the flavonoids and play an important role in plant adaption to environmental stress^14^. Anthocyanins are synthesized in the endoplasmic reticulum and transported to accumulate in the vacuoles of a wide range of cells and tissues in both the vegetative and reproductive organs of plants^15^. The synthesis of anthocyanins in plants is controlled by structural genes and can be divided into four stages^16,17^, with *CHS* and *UFGT* playing a critical role in this metabolic pathway. Shoji et al.^18^ isolated the genes *CHS*, *F3H*, *DFR*, *ANS* and *UFGT* from red lettuce and showed that treatment of red lettuce with blue light spectrum enhanced anthocyanin biosynthesis, with the *CHS* and *UFGT* genes being most associated with anthocyanin accumulation.

Several traits affecting photosynthesis are influenced by light quality, with blue and red light playing the most important role^19^. Hogewoning et al.^20^ reported that increasing blue light increased stomatal conductance (*g*_s_) and the photosynthetic rate (*P*_N_) in cucumber plants. The increase in photosynthesis under the influence of blue light treatment also include effects of a higher chlorophyll a/b ratio^21^ and a higher content of ribulose-1,5-bisphosphate carboxylase/oxygenase (Rubisco)^22^. Although reducing blue light during the growth phase of lettuce decreased the photosynthetic rate more than red light, applying more than 10% blue light to lettuce plants increased the photosynthetic rate^23^. Roni et al.^24^ found that Eustoma plants grown under blue light had higher *P*_N_, *g*_s_ and the transpiration rate (*E*).

Here, an investigation into the effect of different LED spectra and different methods of nutrient solution replacement on biochemical properties, photosynthesis and expression of the Rubisco and anthocyanin biosynthesis pathway genes in two lettuce cultivars was described. The aim of the study was to determine which model of nutrient solution management best contributes to the improvement of these characteristics and allows the avoidance of stress imposed by excessive ion accumulation. The hypothesis was tested that the use of different light spectra may not only improve plant performance but also reduce the negative effect of stress. Most experiments reported so far are limited to the effect of some light spectra on plant growth and development, with fewer studies examining biochemical traits in plants.

## Materials and methods

### Plant material and growth conditions

This experiment was conducted in the plant experiment factory of Vali-e-Asr University in 2020. Seeds of two lettuce varieties (Lollo Rossa and Lollo Bionda) obtained from Sepahan Rooyesh Isfahan and Rijk Zwaan Co. were planted in a seed tray filled with perlite medium. After the four-leaf stage, the seedlings were planted in small plastic pots with perlite medium. These small pots were placed in the holes of the floating systems. After transferring the plants to the floating systems, the Resh nutrient solution formulated for lettuce^25^ (EC: 1.2 ds m^−1^, pH: 6.5) was used. The floating system consisted of 24 square plastic containers measuring 25 × 30 × 30 cm, each floating on a styrofoam measuring 25 × 30 × 30 cm, and four plants planted in each container. All culture containers were connected to the air pump (HAILA, model: ACO-388 D) through holes and the nutrient solution was continuously aerated. Then, 21-day-old seedlings were transferred into holes of Styrofoam. After transplanting, the seedlings were irrigated with a modified Resh nutrient solution containing: 5 mM KNO_3_, 5 mM Ca(NO_3_)_2_, 2 mM MgSO_4_, 1 mM KH_2_PO_4_, 7 μM MnCl_2_, 0.7 μM ZnSO_4_, 0.8 μM CuSO_4_, 0.8 μM Na_2_MoO_4_, 25 μM Fe-EDDHA, and 2 μM H_3_BO_3_. The nutrient solution (pH 6.7 ± 0.1, EC 2.1) was renewed every week. Deionised water was used for nutrient preparation. After reaching the four-leaf stage, the lettuce seedlings were supplied with nutrient solutions or the following 40 days, replaced in three different ways (complete replacement, partial replacement based on the EC value and partial replacement according to the needs of the plant).

In the complete replacement, the nutrient solution was replaced weekly. In the partial replacement according to EC, the EC value of the nutrient solution was adjusted to 1.2 dS m^−1^ by adding predetermined amounts of potassium sulphate, calcium nitrate, magnesium sulphate, potassium dihydrogen phosphate and the half-strength Resh micronutrient solution every 48 h. The replacement of the nutrient solution according to the needs of the plants based on previous reports on tomato^26^ and lettuce and spinach plants^27^ cultivated in a closed hydroponic system. In this method (developed by Roosta), potassium nitrate was used at the same concentration as in the original solution, but the addition of calcium nitrate, magnesium sulphate and potassium dihydrogen phosphate was reduced by three quarters and the microelements (iron, zinc, copper and manganese) were reduced by half and added every two days based on the volume of water added to the plastic containers with the plants. For example, if the lettuce plant absorbed one litre of the solution in two days, one litre of distilled water was first added to the plant container to compensate.

The amount of concentrated solution of different fertilisers needed for this volume was determined on the basis of the original formula, and concentrated solutions in the ratio given above were used instead of the complete solutions. The reason for this was to prevent the accumulation of elements such as magnesium, calcium, phosphorus, and even microelements in the solution and to avoid their toxicity to the plant, as their absorption rate is lower than that of potassium and nitrate. During the experiment, the concentrations of nitrogen (Kjeldahl), calcium and magnesium (by titration), potassium and sodium (flame photometer), phosphorus (spectrophotometer), iron, manganese, copper and zinc (atomic absorption)^28^ in the nutrient solution were determined weekly. Plants were grown at a fully controlled temperature of 25/15 (day/night) and a relative humidity of 50 ± 10%.

### LED tubes and the light treatments

**Figure 1:**
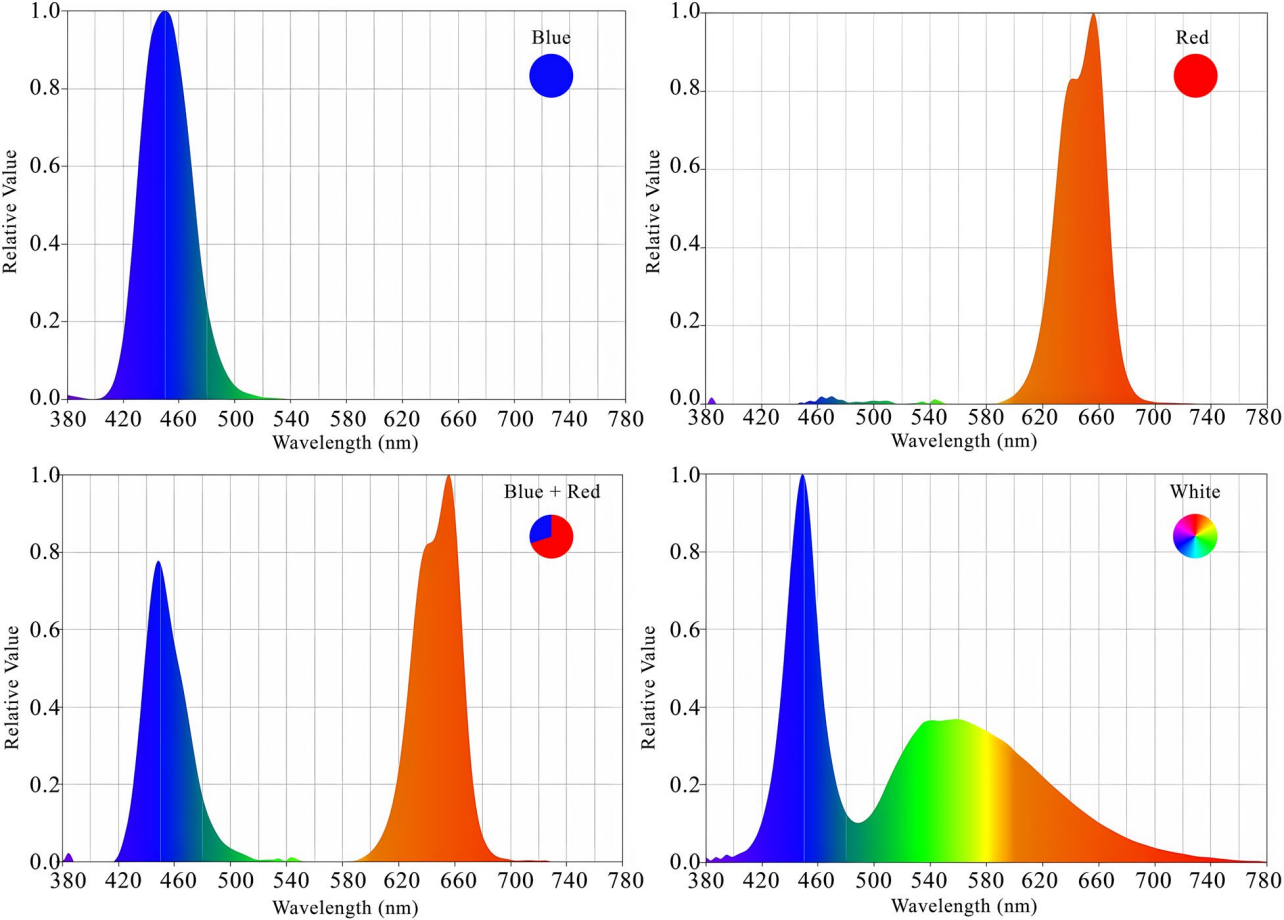
Relative distribution of different LED spectra (red, red/blue 3:1, blue and white) used during the experiment.

Lettuce plants were grown under 24 W LED lamps (Parto Roshd Novin Company, Iran) with different spectral ranges: red (R, with peak at 656 nm), red/blue (3:1; R:B, with peak 656 nm), blue (B, with peak at 450 nm) and white (W, with peak at 449 nm). The photosynthetic photon flux density (PPFD) was 215 ± 5 µmol m^−2^ s^−1^ in all treatments (Fig. 1 and Table 1). The photoperiod of 16/8 h (day/night) was maintained. The LED light systems were placed 30 cm above each individual plant.

**Table 1:** Characteristics of LEDs used in this experiment.

Manufacture company	Efficiency	N. of LEDs	Light coverage area	Power consumption	Lens type	Input Voltage	DC Voltage	Output Current	Output Frequency
Iran Grow Light	90%	24	40 cm × 100 cm	24 × 3W	90 °	AC100-260 V	54–84 V	600 mA ± 5%	50/60 Hz

### Proline measurement

Ninhydrin reagent and the method developed by Paquin and Lechasseur^29^ were used to measure proline. Half a gram of fully developed leaves was homogenised in 5 mL of 95% ethanol in a Chinese mortar and the resulting solution was transferred into a Falcon tube. The solution was centrifuged at 3500 rpm for 10 min and the resulting supernatant was used for spectrophotometric (model T80 UV/VIS Spectrometer PG Instruments Ltd) determination of proline at a wavelength of 515 nm. Standards were prepared with L-proline at: 0, 31.25, 62.5, 125, 250 and 500 (mg L^−1^) concentrations.

### Total soluble sugars

Soluble sugars were measured according to the method of Irigoyen et al.^30^. For this purpose, 0.1 mL of the extract prepared in ethanol (alcoholic extract for proline determination) was mixed with 3 mL of freshly prepared anthrone (200 mg anthrone in 100 mL 72% sulphuric acid) and then incubated in a hot water bath for 10 min. After cooling, the absorbance of the samples was measured at a wavelength of 625 nm.

### Soluble proteins

The method of Bradford et al.^31^ was used to measure the proteins. 0.1 mL of protein extract and 5 mL of the Bradford reagent were added to the test tubes, vortexed briefly and the absorbance read at a wavelength of 595 nm using bovine serum albumin as a standard.

### Determination of anthocyanins

The content of anthocyanins was measured according to the method of Nogués and Baker^32^. One gram of fresh leaf tissue was homogenised in 10 mL of acidic methanol and centrifuged at 3500 rpm. Absorbance was measured in the samples at 530 and 657 nm.

### Total phenolic compounds

The method of Matta et al.^33^ was used to measure the total phenolic compound content. In this method, 0.1 g of leaf tissue was boiled in 10 mL of 80% ethanol and the sample was centrifuged at 3500 rpm. Finally, ithe content was determined with a spectrophotometer at a wavelength of 640 nm. The standard curve was prepared catechol.

### DPPH radical scavenging activity

The measurement of radical scavenging activity in the plant tissue extracts was performed according to the method of Barros et al.^34^. The reduction of DPPH radicals was determined by measuring the absorbance at 517 nm. The radical scavenging activity was calculated as a percentage of DPPH discolouration using the following equation:

$${\text{DPPH}}\,{\text{radical}}\,{\text{scavenging}}\,\% = [({\text{A}}0{-}{\text{A}}1)/{\text{A}}0] \times 100$$

where A0 is the absorbance of the DPPH solution and A1 is the absorbance of the sample.

## Antioxidant enzyme activity

### Preparation of the extract

Half a gram of the fresh leaf tissue was homogenised in three to five millilitres of ice-cold 50 mM potassium phosphate sample buffer (pH 7.2). The homogenate was immediately centrifuged at 4000 rpm for 20–30 min, at four degrees Celsius. The resulting supernatant was collected and stored at − 20 °C until further analysis.

### Peroxidase (POD) activity assay

The enzymatic activity of POD (EC 1.11.1.7) was determined by the oxidation rate of guaiacol to tetraguaiacol according to Plewa et al.^35^. In this method, three millilitres of the reaction mixture contained 2.77 mL of 50 mM potassium phosphate buffer (pH 7.0), 100 µL of 1% hydrogen peroxide, 100 µL of 4% guaiacol, and 30 µL of the enzyme extract. The POD activity was measured following the increase in absorbance at 470 nm for 3 min with an extinction coefficient of 25.5 mM cm^−1^.

### Catalase (CAT) activity assay

CAT (EC 1.11.1.6) activity was measured as previously described by Dhindsa and Motowe^36^. The reaction mixture contained 10 mM H_2_O_2_ in 50 mM potassium phosphate buffer (pH 7.0) and 200 µL enzyme extract in a total volume of three millilitres. The decomposition of hydrogen peroxide was monitored spectrophotometrically by the decrease in absorbance at 240 nm for 3 min (extinction coefficient 28 mM cm^−1^) and expressed per mg of protein.

### Superoxide dismutase (SOD) activity assay

The method of Blanchamp and Fridovitch^37^ was used to measure SOD (EC 1.15.1.1) activity. This measurement was based on the ability of the enzyme to stop the photochemical reduction of nitroblue tetrazolium (NBT) by superoxide radicals in the presence of riboflavin in the light. Fifty microlitres of the extract was mixed with one millilitre of the SOD reaction solution, containing 800 µL 50 mM potassium phosphate buffer (pH 7.8), 50 µL 75 μM NBT, 50 µL 13 mM L-methionine, 50 µL 0.1 mM EDTA, and 50 µL 2 μM riboflavin. The absorbance of thesamples was measured at a wavelength of 560 nm.

### Leaf gas exchange

Photosynthetic parameters were measured using an Infra-Red Gas Analyser (LCi Ultra Compact, ADC BioScientific Ltd, Herts, UK). One fully expanded, intact leaf was selected from each replicate and placed in a leaf cuvette of the instrument. After 6 min to achieve steady-state conditions, net photosynthetic rate (*P*_*N*_; μmol m^−2^ mol^−1^), transpiration rate (*E*; mol H_2_O m^−2^ s^−1^), stomatal conductance (g_s_; mol m^−2^ s^−1^) and intercellular CO_2_ concentration (*C*_*i*_; μmol CO_2_ mol^−1^) were recorded^38^. 

### Analysis of the Rubisco and anthocyanin pathway gene expression

Total RNA was extracted in triplicate from leaves four weeks after transplanting and treatment with LED spectra. Leaf samples were immediately frozen in liquid nitrogen and stored at − 80 °C until further use. Samples were ground in liquid nitrogen with a pestle and mortar and RNA was extracted using Column RNA isolation kit (DENAzist Asia, Ferdowsi University of Mashhad, Mashhad, Iran). The preparation of the stock and working solution of primers (oligonucleotides) was done according to the manufacturer’s guidelines (DENAzist Asia). cDNA was synthesised using the RevertAid first strand cDNA synthesis kit (Thermo Scientific™, Waltham, MA USA). Any DNA contamination was removed using DNaseI Digestion Set (Sigma-Aldrich). The purity and quantity of RNA was measured spectrophotometrically (Nanodrop™ Microvolume UV-Vis Spectrophotometer, Thermo Scientific™) and assessed by electrophoresis on a 1% (w/v) agarose gel using 200 ng of RNA. The A_260_/A_280_ and A260/A230 ratios measured in the samples were higher than 1.8. Real-time quantitative PCR (qRT-PCR, Thermo Fisher, USA) was performed to analyse the expression levels of the Rubisco small subunit gene (rbcS) and anthocyanin biosynthesis genes in the leaf tissue. Gene-specific primers were designed using Primer-BLAST (NCBI, https://www.ncbi.nlm.nih.gov/tools/primer-blast/) (Table 2). qRT-PCR was performed in triplicate using SYBR Green 2X Real-Time PCR MasterMix including SYBR Green 1, Low ROX (BioFACTTM, Korea). Actin (AB359898) was used as an internal control. The 2^−ΔΔCT^ method was used to calculate expression levels as previously described^39^.

**Table 2:** Specific primers selected for small subunit Rubisco gene and anthocyanin biosynthesis pathway genes by Real-time PCR analysis.

Gene name	Accession number	Product length	Name	Primer sequence (5ʹ–3ʹ)
Rubisco	AF162210.1	835 bp	small subunit-For	TCTAACGTCGCTTTCCCAGT
			small subunit-Rev	GTCGGACAATGGTGGTAGGT
Actin	AB359898	113 bp	LsACT-F01	TGGTAGGTATGGGCCAGAAA
			LsACT-R01	GTCATCCCAGTTGCTCACAA
CHS	AB525909	169 bp	LsCHS-F02	GGAGGTGGGGCTAACTTTTC
			LsCHS-R02	GAGCTCCACCTGGTCCAATA
UFGT	AB525911	203 bp	LsUFGT-F02	AAGAGACCAGAACCCCGTTT
			LsUFGT-R02	AGCTCCAATGCTCTCCGATA

### Experimental design and data analysis

The experiment was a completely randomised design with three factors in three replicates in factorial form and two plants of each variety in a plastic container. SAS software version 9.4 was used to analyse all data (SA stitute, Cary, NC, USA). All data were statistically analysed using the two-way ANOVA. When analysis of variance indicated significant treatment effects, significant mean differences (P<0.05) were calculated with the Duncan Multiple Range Test. Range tests identify homogeneous subsets of means that do not differ from each other. A correlation plot was drawn with Origin Pro software version 2021. The graphs were made using Excel 2013 (Microsoft, Redmont, WA, USA). The results were expressed as mean values and their standard errors (SE) using MS Excel software.

### Statement of compliance

The authors confirm that all the experimental research, including the collection of plant material, complied with relevant institutional, national, and international guidelines and legislation.

## Results

### Proline content

The results showed that the proline concentration in the lettuce leaves was affected by the variety, the light quality and the method used to replace the nutrient solution, as well as by the interaction of these factors (Table 3). The combination of red and blue LED light increased the proline content in both lettuce cultivars grown under the complete nutrient solution replacement and the method based on the EC value compared to the control treatment (white light spectra and complete nutrient solution replacement). The proline content was also elevated in Lollo rossa and Lollo Bionda in response to the red and red/blue spectra, respectively, fed according to the needs of the plants (Table 4).

**Table 3:** The effect of different light qualities and three replacement methods of nutrient solution on physiological characteristics of two varieties of lettuce in floating hydroponics.

Source of Variation	Df	Proline	Soluble sugar	Soluble protein	Phenolic compounds
Variety (V)	1	0.044*	3.04**	0.21ns	0.0001ns
Light (L)	3	0.55**	12.6**	0.95**	10.9**
Nutrient solution (N)	2	0.05**	0.07ns	0.02ns	1.20**
V*L	3	0.26**	13.4**	0.76**	4.33**
V*N	2	0.36**	16.3**	1.29**	12.24**
L*N	6	0.54**	7.49**	0.56**	8.25**
V*L*N	6	0.22**	4.51**	0.25*	4.48**
Experimental Error	48	0.008	0.52	0.09	0.019
Coefficient Variance (%)	–	4.3	4.04	6.62	1.62

^ns^, * and ** indicate non-significance, significant at 5% and 1% probability level (Duncan’s Test), respectively.

**Table 4:** The interactive effects of lettuce variety, light quality and three replacement methods of nutrient solution on the proline and total soluble sugar content in lettuce plants.

Variety	Light	Proline (mg g^−1^ FW)			Total soluble sugars (mg g^−1^ FW)		
		Complete replacement	Based on EC	Based on plant needs	Complete replacement	Based on EC	Based on plant needs
Lollo Rossa	White	2.39cde	2.54b	2.15fgh	18.01d-g	16.68hi	15.01j
	Blue	2.12gh	1.91ijk	1.74klm	17.67e-h	17.57fgh	15.8ij
	Red/Blue	2.88a	2.58b	1.88i-l	20.03ab	21.09a	19.09bcd
	Red	1.57n	2.09gh	2.29ef	18.98b-e	21.2a	15.39ij
Lollo Bionda	White	2.10gh	1.65mn	2hi	16.6hi	18.35c-f	16.7ghi
	Blue	2.33e	2.36de	1.92ij	19.44bc	19.33bcd	16.5hi
	Red/Blue	2.63b	2.51bcd	2.22efg	17.51fgh	17.4fgh	18.4c-f
	Red	1.83jkl	2.26efg	1.72lmn	17.49fgh	18.56c-f	15.7ij

Values are means ± SE of three replicates. Different letters in each column show significant differences at *P* ≤ 0.05 (Duncan’s Test).

### Total soluble sugars

Soluble sugar content in the lettuce plants was influenced by lettuce variety and light quality, interactions between variety and light quality, light quality and nutrient replacement methods, and interactions between these factors (Table 3). In the Lollo Rossa cultivar, the use of red/blue and monochromatic red light in the EC-based replacement method and red/blue LED light in the complete replacement had the most pronounced effect on soluble sugar content, with an increase of up to 18% observed in compared to the control plants. In Lollo Bionda, however, the application of blue light increased the amount of total soluble sugars by 17 and 16% in plants grown under the complete replacement and the replacement method based on EC, respectively. When the same plants were supplemented with nutrients according to the needs of plants, the combination of red and blue light increased the soluble sugar content by 11% (Table 4).

### Soluble protein

The results showed that soluble protein concentration was influenced by the effects of light quality, the interaction between variety and light quality, the interaction between light quality and replacement methods of nutrient solution and the interaction of these three factors (Table 3). The red/blue and red LED light increased the amount of soluble protein in the Lollo Rossa variety by 41.2 and 31.5%, respectively, in the replacement method based on the EC value and by 31.3 and 27.1%, respectively, in the complete replacement method (Table 5). Moreover, the application of the red/blue and white light spectrum had a positive effect on the protein content when this variety was grown according to the needs of the plant. At the same time, the use of the blue light spectrum and both the EC-based replacement and the complete replacement method and the combination of red and blue LED light and replacement method based on the needs of the plant resulted in up to 23% increase of the total protein content in the leaf tissue of Lollo Bionda (Table 5).

**Table 5:** The interactive effects of lettuce variety, light quality and three replacement methods of nutrient solution on soluble protein and phenol compounds in lettuce plants.

Variety	Light	Soluble protein (mg g^−1^ FW)			Phenolic compounds (mg g^−1^ FW)		
		Complete replacement	Based on EC	Based on plant needs	Complete replacement	Based on EC	Based on plant needs
Lollo Rossa	White	4.02g	4.48d-g	4.84b-f	8.47jk	9.91e	7.62l
	Blue	4.49d-g	4.73c-f	4.26efg	8.75i	7.87l	6.55n
	Red/Blue	5.28ab	5.68a	4.85b-e	7.67l	11.37a	11.01b
	Red	5.11abc	5.29ab	4.04g	5.90o	8.52ijk	10.24d
Lollo Bionda	White	4.23fg	4.93bcd	4.24efg	10.50c	6.49n	8.66ij
	Blue	4.94bcd	5.20abc	4.43d-g	10.17d	9.15h	7.86l
	Red/Blue	4.7c-f	4.68c-f	4.97bcd	9.45fg	9.62f	9.32gh
	Red	4.44d-g	4.71c-f	4.47d-g	7.21m	8.39k	7.08n

Values are means ± SE of three replicates. Different letters in each column show significant differences at *P* ≤ 0.05 (Duncan’s Test).

### Phenolic compounds

Based on the results, it was found that the concentration of phenolic compounds was affected by light quality, nutrient solution replacement method, interaction between variety and light quality, interaction between variety and nutrient solution replacement method, interaction between light and nutrient solution replacement method, and the interaction of these three factors (Table 3). As shown, the Lollo Rossa variety treated with red/blue LED light and supplied with nutrients based on both the EC value and the plant requirement had 34.2 and 30% higher phenolic compounds content, respectively, than the control plants (Table 5). In plants fed according to plant requirement, the monochromatic red light also showed a positive effect on this parameter, with an increase of 21%. In contrast, in the Lollo Bionda, variety the use of white and blue LED light resulted in increased concentrations of total phenolic compounds when the nutrient solution was completely replaced (Table 5).

### Antioxidant activity

DPPH radical scavenging activity was affected by light quality, method of nutrient solution replacement, interaction between variety and light quality, interaction between variety and nutrient solution replacement method, interaction between light and replacement method, and the interaction of these three factors (Table 6). It was found that combination of red and blue LED light increased antioxidant activity in leaf tissue by 30 and 11%, respectively, in Lollo Rossa plants grown under the replacement method based on plant needs and EC value compared to the control (Table 7). In Lollo Bionda, plants grown under the white LED light and complete nutrient solution replacement showed the highest DPPH radical scavenging activity in this cultivar, with two other replacement methods contributing to decrease in this trait (Table 7).

**Table 6:** The effect of different qualities of light and three methods of replacement of nutrient solution on DPPH radical scavenging activity and enzymes activity of two varieties of lettuce in floating hydroponics.

Source of variation	Df	DPPH radical scavenging activity	Guaiacol peroxidase enzyme activity	Catalase enzyme activity	Superoxide dismutase enzyme activity
Variety (V)	1	0.0001ns	2234**	8.69*	534**
Light (L)	3	176**	24,004**	275**	10,162**
Nutrient solution (N)	2	26.5**	2361**	27.09**	118ns
V*L	3	194**	6937**	63.9**	1990**
V*N	2	142**	20,389**	154**	7233**
L*N	6	183**	18,891**	255**	11,573**
V*L*N	6	82.2**	9500**	135**	2339**
Experimental Error	48	1.91	80.2	1.46	52.8
Coefficient Variance (%)	–	3.28	3.28	4.0	3.06

ns, * and ** indicate non-significance, significant at 5% and 1% probability level (Duncan’s Test), respectively.

### Guaiacol peroxidase activity

The enzymatic activity of guaiacol peroxidase activity was affected by plant variety, light quality, method of nutrient solution replacement, and the interaction of these three factors (Table 6). The application of red/blue LED light in the complete replacement method and the EC-based replacement method resulted in a 40.2 and 36.5% increase in guaiacol peroxidase activity in Lollo Rossa, respectively (Table 7). In the Lollo Bionda cultivar, plants grown under the complete replacement method showed the highest enzyme activity when exposed to both white and blue LED light. For the other two replacement methods applied to Lollo Bionda, the combination of red and blue light spectra maintained higher activities of guaiacol peroxidase (Table 7).

**Table 7:** The interactive effects of lettuce variety, light quality and three replacement methods of nutrient solution on DPPH radical scavenging activity and guaiacol peroxidase enzyme activity in lettuce plants.

Variety	Light	Antioxidant activity (% inhibition of DPPH radical)			Guaiacol peroxidase enzyme activity (unit enzyme activity ∙ mg^−1^ protein)		
		Complete replacement	Based on EC	Based on plant needs	Complete replacement	Based on EC	Based on plant needs
Lollo Rossa	White	44.1d	51.6b	39.7fgh	276e	329c	235fg
	Blue	37.7ghi	33.9j	34.07j	269e	229g	176j
	Red/Blue	39.8fg	49.03c	57.4a	387a	377a	209h
	Red	30.66k	44.3d	44.1d	321c	247f	149k
Lollo Bionda	White	52.9b	33.7j	37.3hi	359b	179j	275e
	Blue	45.2d	47.6c	40.89f.	350b	305d	229g
	Red/Blue	40.7f.	41.4ef	48.5c	289d	323c	331c
	Red	37.4ghi	43.7de	36.8i	211h	282e	194i

Values are means ± SE of three replicates. Different letters in each column show significant differences at *P* ≤ 0.05 (Duncan’s test).

### Catalase activity

Results from ANOVA showed that catalase activity was affected by variety, light quality, nutrient solution replacement method, and the interaction of these three factors (Table 6). It was found that red/blue light increased catalase activity in the Lollo Rossa cultivar by 10.2 and 20% when plants were grown using the EC-based nutrient solution replacement method and the complete replacement method, respectively (Table 8). However, in Lollo Bionda variety, the plants fed with the complete replacement method under white LED light and with the EC-based replacement method under red/blue LED light showed the highest enzymatic activity (Table 8).

**Table 8:** The interactive effects of lettuce variety, light quality and three replacement methods of nutrient solution on catalase enzyme activity and superoxide dismutase enzyme activity in lettuce plants.

Variety	Light	Catalase enzyme activity (unit enzyme activity mg^−1^ protein)			Superoxide dismutase enzyme activity (unit enzyme activity mg^−1^ protein)		
		Complete replacement	Based on EC	Based on plant needs	Complete replacement	Based on EC	Based on plant needs
Lollo Rossa	White	35.2cde	34.9cde	31.6gh	269e	258ef	215ij
	Blue	32.3fgh	20.4k-n	29.10i	275ef	206jk	199kl
	Red/Blue	38.8b	42.2a	21.8kl	309b	323a	191l
	Red	24.2j	38.01b	18.2n	239gh	247fg	159n
Lollo Bionda	White	38.7b	30.5hi	19.13mn	296c	216ij	167mn
	Blue	35.5cd	22.2k	34.8cde	283d	237gh	219ij
	Red/Blue	33.9def	36.9bc	32.0fgh	270e	282d	226hi
	Red	33.2efg	20lmn	21.2klm	226hi	209jk	175m

Values are means ± SE of three replicates. Different letters in each column show significant differences at *P* ≤ 0.05 (Duncan’s test).

### Superoxide dismutase activity

Superoxide dismutase activity was affected by variety, light quality, interaction of variety and light quality, interaction of variety and replacement method of nutrient solution, interaction between light and nutrient solution replacement method, and the interaction of these three factors (Table 6). The combination of red and blue LED light significantly induced superoxide dismutase activity in the leaves of Lollo Rossa—it was increased by 15 and 20% compared to the control conditions when the complete replacement method and the EC-based replacement method were used, respectively. In contrast, in the Lollo Bionda variety, the use of blue light in the complete replacement method and red/blue LED light in the EC-based replacement method resulted in the highest enzyme activity, which was close to the control level (Table 8).

### Anthocyanins concentration

**Figure 2:**
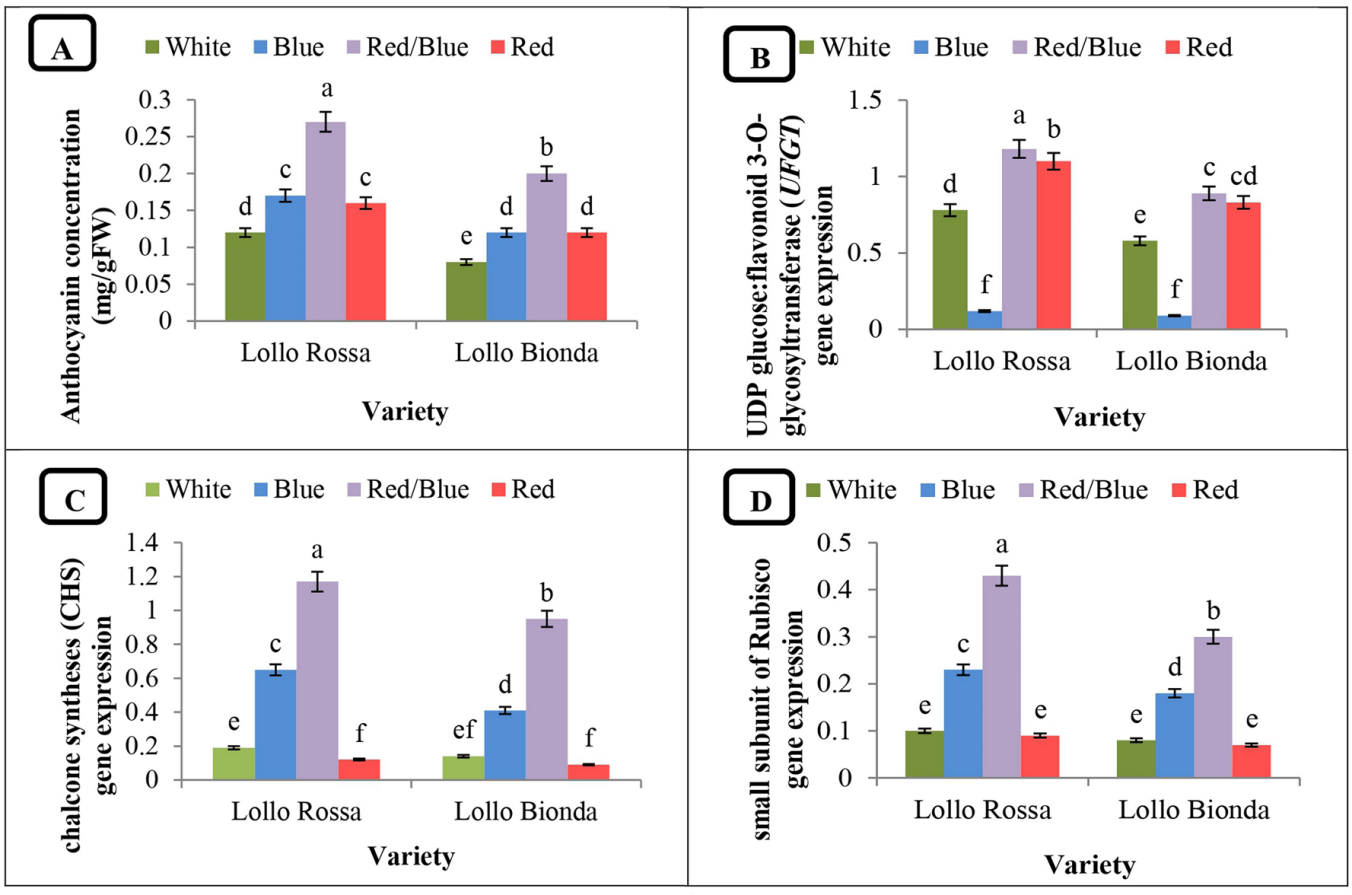
The interactive effect of lettuce variety and LED light quality on (**A**) anthocyanin concentration, (**B**) UDP glucose:flavonoid 3-O-glycosyltransferase (*UFGT*) gene expression, (**C**) chalcone synthase (*CHS*) gene expression and (**D**) Rubisco small subunit gene expression in lettuce plants.

Anthocyanin concentration was affected by variety, light quality and the interaction of variety and light quality (Table 9). It was found that irradiation with red/blue LED light resulted in a more than twofold increase of anthocyanin content in both varieties in compared to control plants, however, in Lollo Bionda this never reached the high levels characteristic of Lollo Rossa (Fig. 2). Importantly, the separate application of both monochromatic spectra was able to increase anthocyanin concentration compared to the control treatment (white light). However, in both varieties, plants treated with red and blue light did not differ significantly in anthocyanin content (Fig. 2A).

**Table 9:** The effect of variety and LED light quality on anthocyanins concentration and the expression of genes of anthocyanin biosynthesis pathway and the small sub-section of Rubisco.

Source of Variation	DF	Anthocyanins concentration	*UFGT*	*CHS*	Small subunit of rubisco
Variety (V)	1	0.014**	0.24**	0.25**	0.04**
Light (L)	3	0.018**	1.06**	3.77**	0.73**
L*V	3	0.0003*	0.02**	0.07**	0.01**
Experimental Error	16	0.001	0.001	0.03	0.0003
Coefficient Variance (%)	–	5.36	5.36	4.26	6.19

* and ** indicate significance at the 5 and 1 percent probability level (Duncan’s test), respectively.

### UDP glucose: flavonoid 3-O-glycosyltransferase (*UFGT*) gene expression

The expression level of the *UFGT* gene was affected by variety, light spectrum and the interaction of lettuce variety and LED light quality (Table 9). It was found that treatment with the red/blue LED light and the red monochromatic LED light resulted in increased (by 50% and 40%, respectively) expression level of the *UFGT* gene in both lettuce cultivars compared to control plants, while the lowest *UFGT* expression was observed in lettuce plants grown under the blue spectrum (Fig. 2B).

### Chalcone synthase (*CHS*) gene expression

Chalcone synthase gene expression was affected by lettuce variety, light quality and the interaction of these factors (Table 9). As shown, treatment of lettuce plants with the red/blue light spectrum and with monochromatic blue light in a significant increase in *CHS* expression compared with control plants—6.2- and 3.3-fold in Lollo Rossa and 6.2- and 2.7-fold in Lollo Bionda, respectively (Fig. 2C). The lowest gene expression was observed in the plants grown under monochromatic red light (Fig. 2C).

### Small Rubisco subunit gene expression

Expression of the Rubisco small subunit gene was affected by lettuce variety, light quality, and the interaction of these factors (Table 9). During the experiment, the plants treated with the combination of red and blue LED light and monochromatic blue light showed the highest Rubisco gene expression (Fig. 2D). Lollo Rossa responded to the red/blue and blue lights with a 4.3- and 2.3-fold increase in Rubisco expression, respectively, while it was increased 3.7- and 2.2-fold in Lollo Bionada compared with control plants. The expression levels determined in the plants treated with monochromatic red light were significantly lower and close to the control values (Fig. 2D).

### Net photosynthetic rate (*P*_N_)

**Figure 3:**
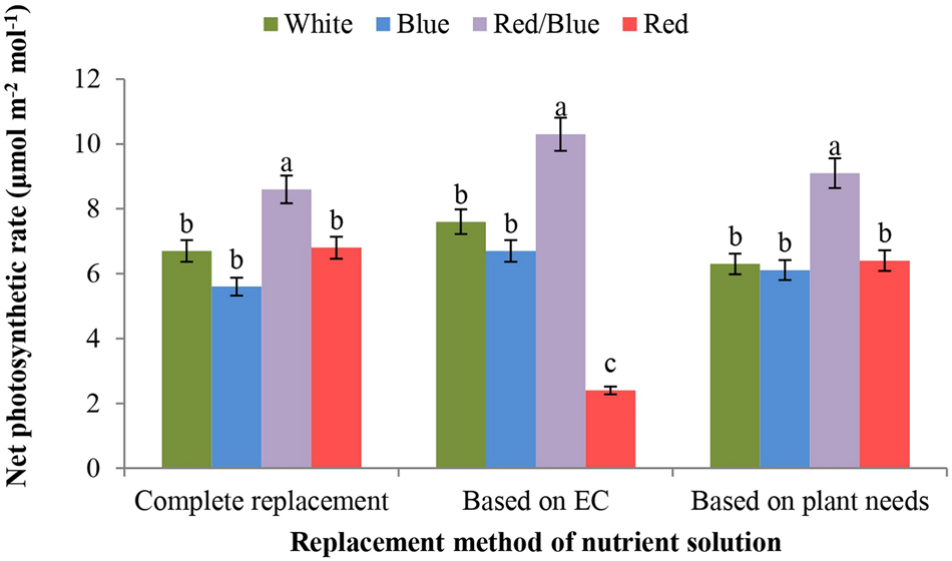
The interactive effect of nutrient solution replacement method and LED light quality on net photosynthetic rate in lettuce plants.

The net photosynthetic rate was affected by the light spectrum and the interaction of light quality and nutrient solution replacement method (Table 10). The use of combined red and blue light in all three nutrient solution replacement methods resulted in a 30–45% increase in net photosynthesis of lettuce cultivars compared to control conditions. The other light spectra did not show statistically significant differences from each other, except for the red LED light treatment in plants fed according to the EC value (Fig. 3).

**Table 10:** The effect of different light qualities and three replacement methods of nutrient solution on gas exchange characteristics of two varieties of lettuce in floating hydroponics.

Source of Variation	Df	*P*_N_	C_*i*_	*E*	*g*_*s*_
Variety (V)	1	0.20ns	826ns	2.55ns	0.007ns
Light (L)	3	30.8*	2305*	2.6*	0.025**
Nutrient solution (N)	2	3.74ns	710ns	0.17ns	0.002ns
V*L	3	5.99ns	672ns	1.8ns	0.034**
V*N	2	17.2ns	324ns	9.1**	0.034**
L*N	6	50.1**	2742**	5.48**	0.067**
V*L*N	6	18.1ns	966ns	5.05**	0.017**
Experimental Error	48	10.35	700	0.80	0.002
Coefficient Variance (%)	–	4.59	7.98	20.8	23.5

ns, * and ** indicate non-significance, significant at 5% and 1% probability level (Duncan’s Test), respectively.

### Intercellular CO_2_ concentration (*C*_i_)

**Figure 4:**
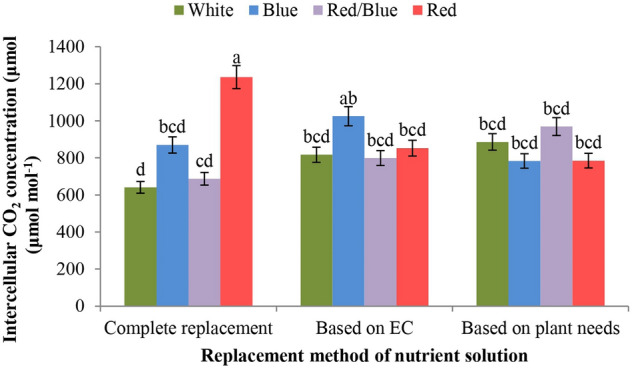
The interactive effect of nutrient solution replacement method and LED light quality on intercellular CO_2_ concentration in lettuce plants.

The *C*_i_ parameter was affected only by the light spectrum and the interaction of light and nutrient solution replacement method (Table 10). It was found that the plants grown under complete replacement and EC-based method exposed to monochromatic red and blue light, respectively, showed higher *C*_i_ than the plants exposed to white light (control). Other light spectra showed no statistically significant differences among all three replace methods (Fig. 4).

### Transpiration rate (*E*)

Based on the results, it was found that transpiration rate was affected by light spectrum, interaction of light spectrum and replacement method of nutrient solution, interaction of variety and light spectrum, variety and replacement method of nutrient solution, light and replacement method of nutrient solution, and the interaction of these three factors (Table 10). The application of monochromatic blue and white light and the replacement method based on the needs of the plants in the Lollo Bionda cultivar increased the transpiration rate by 82.7 and 65.5%, respectively, in relation to the control. Overall, the transpiration rate was higher all light spectra applied to lettuce varieties fed based on the needs of plants in comparison to the control treatment (Table 11).

**Table 11:** The interactive effects of lettuce variety, light quality and three replacement methods of nutrient solution on the transpiration rate and stomatal conductance in lettuce plants.

Variety	Light	Transpiration rate (mol (H_2_O) m^−2^ s^−1^) (E)			Stomatal conductance (mol H_2_O m^−2^ s^−1^) (gs)		
		Complete replacement	Based on EC	Based on plant needs	Complete replacement	Based on EC	Based on plant needs
Lollo Rossa	White	3.08 efgh	3.92 cdef	5.46 abc	0.12def	0.18def	0.21cde
	Blue	4.14 bcdef	4.44 bcdef	5.22 abcd	0.16def	0.32b	0.33b
	Red/Blue	2.91 fgh	4.52 bcdef	5.43 abc	0.09f	0.31b	0.40ab
	Red	4.52 bcdef	3.96 cdef	5.37 abc	0.18def	0.13def	0.34ab
Lollo Bionda	White	3.54 defg	4.01 cdef	5.86 ab	0.22cd	0.15def	0.40ab
	Blue	4.01 cdef	1.87 h	6.47 a	0.113ef	0.31b	0.43a
	Red/Blue	3.02 efgh	1.94 gh	4.70 bcde	0.116def	0.113ef	0.21de
	Red	3.57 defg	4.15 bcdef	4.77 abcde	0.116def	0.16def	0.22cd

Values are means ± SE of three replicates. Different letters in each column show significant differences at *P* ≤ 0.05 (Duncan’s test).

### Stomatal conductance (*g*_s_)

Stomatal conductance was affected by light spectrum, interaction of light and replacement method of nutrient solution, the interaction of variety and light spectrum, variety and replacement method of nutrient solution, light and replacement method of nutrient solution, and the effects of these three factor (Table 10). The use of the blue and white light spectrum under the replacement method based on plant needs resulted in the highest values of stomatal conductance in the Lollo Bionda cultivar (95.4 and 81.8% higher than the control, respectively). In the Lollo Rossa variety, the red/blue light was the most effective in inducing stomata opening. The stomatal conductance determined under all light spectra was higher in lettuce supplemented according to the needs of the plant than in the control (Table 11).

## Discussion

Light is an essential environmental factor for plants playing an important role in regulating growth, morphology, and metabolism^40^. Proline is a compatible osmolyte component that is usually induced in response to osmotic imbalance in the cytoplasm of cells^41^. Under stress, plants need to maintain intracellular water potential to sustain turgescence and effectively absorb water, with proline facilitating water uptake and turgor pressure stability. Proline is also a highly effective osmoprotectant, improving protein stability and protecting membrane integrity^42^. In the present experiment, the lettuce varieties (Lollo Rossa and Lollo Bionda) treated with red/blue LED light and grown under the complete replacement and EC-based method had the highest proline concentration (Table 4), supporting the role of this osmolyte in preventing dehydration, reducing cellular damage, maintaining osmotic balance and stress tolerance to the plant^43^. The results obtained here are consistent with studies on the responses of a number of plant species to osmotic stress, including studies on model plant *Arabidopsis thaliana*^41^.

Soluble sugars play many important roles in the plant cell, including the functions of key components of energetic and biosynthetic metabolism. Importantly, however, they can also act as compatible osmolytes, restoring osmotic balance, and as protective macromolecules^41^. It has long been known that salinity and a high EC in the nutrient solution lead to a decrease in the content of soluble sugars in the tissues of leafy vegetables^43^. However, it has been shown that different light conditions can modulate the sugar content in plants by influencing the activity of enzymes of sucrose metabolism^44^. Here, the red/blue and the monochromatic red LED spectra in lettuce cultivars grown under the replacement method based on the EC values and the complete replacement resulted in a higher soluble sugar content (Table 4). In the studies by Ding et al.^7^ it was shown that an increase light intensity caused a decrease in soluble sugar content. In contrast, Chen et al.^44^ reported that the combination of red and blue light as well as monochromatic red light can increase the accumulation of soluble sugar in lettuce plants, which is consistent with the results of the present study (Table 4). Our results are further supported by the fact that the 70/30% and 80/20% red/blue combination and 100% red light were described as most effective in increasing soluble sugar content in lettuce plants^45^.

Abiotic stresses, such as salinity and an increased EC in the nutrient solution, can lead to decrease in soluble protein content. This loss may be due to an increase in the rate of protein degradation^46^. This is in agreement with our findings, where the increased accumulation of proline in plants grown under complete replacement and the EC-based method of nutrient solution was clearly correlated with the decreased soluble protein content. This is also confirmed by Liu et al.^47^ who indicated that high light intensity and both high and low EC can reduce soluble protein concentration in lettuce plants. However, it is important to note that the results of the present experiment showed that the red/blue and monochromatic red LED spectrum resulted in higher soluble protein content in lettuce varieties in plants cultivated under the complete nutrient solution replacement and replacement method based on EC, despite the increased proline accumulation (Table 5).

Phenolic compounds are among the most important antioxidants in plant cells whose production is stimulated under the influence of stress^48,49^. In the present experiment, the complete replacement of the nutrient solution in Lollo Bionda and the EC-based replacement method in Lollo Rossa had the highest effect on the total content of phenolic compounds under the combination of red and blue LED light (Table 5), which is in agreement with previous results in turnip plants where an increased EC level in the nutrient solution led to an increased content of phenolic and flavonoid compounds. The induction in the phenolic accumulation could possibly be explained by a modulation of the enzymatic activity of phenylalanine aminolyase—the first enzyme in the phenylpropanoid pathway of phenolic compounds synthesis, by the reduced water potential due to the high EC values in the nutrient solution^7^. It is well known that phenolic compounds can reduce cell membrane damage caused by oxidative stress due to their antioxidant potential in plants^50^. Previously, it was reported that blue and red LED spectra increase the content of flavonoids and phenolic compounds in cucumber and *R. hongnoensis* plants^13,51^.

Antioxidants in plants are important intracellular compounds functioning to prevent the activity of reactive oxygen species (ROS). Previous studies have reported that light quantity and quality can have strong effects on impaired stimulation of PSII or PSI, leading to energy imbalance between photosystems^52^ and increased ROS generation^53^. To scavenge ROS, plants induce antioxidant mechanisms by increasing the levels of non-enzymatic antioxidants and the activity of enzymes such as catalase, superoxide dismutase and peroxidase^54^. Antioxidant enzymes protect important cellular components, therefore assessing their activity is one of the most reliable ways to evaluate the stress resistance of plants^55^. Furthermore, measuring the antioxidant capacity of plants is of particular importance in the context of dietary values^56^. In the present experiment, it was shown that the complete replacement of the nutrient solution and the EC-based replacement method led to an increase in antioxidant enzymes in both lettuce varieties. This is consistent with previous founding in basil and saffron plants, where increasing the concentration of the Hoagland nutrient solution provoked an increase in both enzymatic and non-enzymatic antioxidants^57,58^. The high activity of antioxidant enzymes observed in treatments with high EC levels could be explained by the direct toxic effect of the minerals accumulating at high levels and by a negative effect on water uptake, provoking dehydration symptoms^59^. In the present study, data was also provided that the treatment of the plants with primarily red/blue LED light was able to induce the activity of the antioxidant enzymes and the radical scavenging activity in the cultivated lettuce varieties (Tables 7, 8). Consistent with previous findings^48,50^, there was evidence that the amount of phenolic compounds and antioxidant activity depended on the variety and plant species.

The accumulation of anthocyanins is regulated by transcription factors and stimulated by various environmental factors such as light, low temperatures, drought and salt stress^60^. Different light qualities and intensities are sensed by receptors or signalling factors to be transduced into the downstream transcription factors^61^. Next, enzymes encoded by structural genes (these genes are in turn regulated by transcription factors) synthesise metabolites in response to light. In the present study, the combined application of red LED and blue LED light significantly increased the anthocyanin content in both lettuce varieties compared to other light spectra used during the experiment (Fig. 2). At the same time, the use of the individual monochromatic spectra resulted in a higher anthocyanin concentration compared to the control values determined in the plants grown under the white LED light. However, the plants treated with red LED and blue LED light did not differ significantly in terms of anthocyanin content (Fig. 2). As expected, our results showed that the anthocyanin content was higher in the red-leaved lettuce variety (Lollo Rossa) than in the green-leaved variety (Lollo Bionda). These results are consistent with previous reports where the combination of red and blue LED light increased the accumulation of anthocyanins in the Batavia lettuce variety (*Lactuca sativa* cv. Batavia)^62^.

According to the data from this study, the red/blue LED light increased the expression of the *UFGT* gene in both lettuce cultivars compared to the control (white LED light; Fig. 3). It was also shown that in both lettuce cultivars, the highest expression of the *CHS* gene was observed under the red/blue LED light and the monochromatic blue light spectrum, which was clearly superior to the white LED light (Fig. 4). In general, the expression of the genes *CHS* and *UFGT* determined in this study was higher in the red-leaved lettuce variety (Lollo Rossa) than in the green-leaved variety (Lollo Bionda). This is in line with previous reports indicating that the expression of the genes *CHS* and *UFGT* in red-leaved lettuce is dependent on the ratio between red and blue light^18^. Recently, the combination of red and blue light was shown to stimulate the expression of genes involved in the biosynthesis of secondary metabolites such as anthocyanins and phenols in lettuce^62^. Since the use of monochromatic blue light increased the expression of the *UFGT* gene, while the expression of *CHS* increased under monochromatic red light (Figs. 3, 4), it can be concluded that the combination of red and blue light has a synergistic effect on the production of anthocyanins. More recently, it was pointed out that increasing the amount of blue light increases the anthocyanin content in purple pepper fruits via an increase in anthocyanin biosynthesis, whichis supported by a higher expression of the anthocyanin biosynthesis genes *CaMYB*, *CaCHS*, *CaDFR*, *CaANS* and *CaUFGT*^63^.

Stomata in plants are important channels for water and CO_2_ exchange, which is strictly regulated by light^64^. In this study, the red/blue LED light spectrum had a clear inductive effect on net photosynthesis under all three nutrient solution replacement methods (Fig. 3). At the same time, the lettuce plants treated with monochromatic red and monochromatic blue LED light, respectively, under the complete replacement method and the EC-based method showed the most pronounced effect on intercellular CO_2_ concentration (Fig. 4). The use of the blue light spectrum under the replacement method based on the needs of plant resulted in the highest level of stomatal conductance and transpiration rate in Lollo Bionda cultivar (Table 11), which is consistent with the role of blue light in the cryptochrome- and phototropin-mediated mechanism of stomata opening^64,65^. The control of gas exchange between the leaf and the atmosphere through stomata determines photosynthetic CO_2_ uptake for photosynthesis and transpiration, and thus plant productivity and water use efficiency. The balance between these two processes depends on the responses of stomata to environmental and internal stimuli and on the synchrony of stomata behaviour relative to the CO_2_ demand of the mesophyll^65^.

In the present study, irradiation of the investigated lettuce varieties with red/blue light and the monochromatic blue light resulted in increased expression of the Rubisco small subunit gene (RBCS) compared to the control treatment (white light; Fig. 2D). The combination of red and blue light is a factor that modulates the morphological and photosynthetic characteristics of plants through its effect on leaf anatomy, photosynthetic electron transport and expression of key Calvin cycle enzymes such as Rubisco, fructose-1,6-bisphosphatase (FBP) and glyceraldehyde phosphatase dehydrogenase (GAPDH)^66^. The results of this experiment are consistent with previous findings that the expression of the Rubisco genes RBCS and RBCL increased under red and blue LED light as opposed to white light^67^. More recently, white LED light was reported to increase the expression of the Rubisco activase (RCA) gene in Gerbera plants compared to the combination of red and blue light^68^. Since a negative correlation between the Rubisco activase and Rubisco levels has been reported, we speculate that RBCS expression may decrease in response to upregulated RCA^69^. The observed increase in Rubisco gene expression (Fig. 2D) with the concomitant increase in net photosynthesis (Fig. 3) due to the interaction of red and blue LED light reflects the ability of this light spectrum to enhance CO_2_ assimilation through increased Rubisco activity, resulting in increased biomass production and yields in plants fed according to the nutritional needs.

## Conclusions

In conclusion, it was shown that, in contrast to nutrient replacement based on plant needs, the methods of complete replacement and EC-based replacement increase the amount of compatible osmolytes due to stress conditions resulting from the accumulation of mineral elements in the nutrient solution. The combination of red and blue light was able to reduce the damage caused by osmotic stress by increasing the content of proline and soluble sugar and the activity of antioxidant enzymes. The increased expression of *CHS* and *UFGT* genes observed under combination of red and blue LED light resulted in increased accumulation of anthocyanins in the leaves of red-leaved lettuce cultivar Lollo Rossa. The combination of red and blue LED light and the replacement method based on the needs of the plants are recommended for lettuce cultivars because they have significant positive effects on the biochemical properties and gene expression in anthocyanin synthesis. At the same time, it can prevent the waste of water and nutrients and protect the environment.

## Data Availability

The datasets generated during and/or analysed during the current study are available from the corresponding authors on reasonable request.
